# Steroid receptors and coregulators: Dissemination of sex differences and emerging technologies

**DOI:** 10.1016/j.jbc.2025.108363

**Published:** 2025-02-27

**Authors:** Sally N. Pauss, Evelyn A. Bates, Genesee J. Martinez, Zane T. Bates, Zachary A. Kipp, Cassandra D. Gipson, Terry D. Hinds

**Affiliations:** 1 Drug & Disease Discovery D3 Research Center, Department of Pharmacology and Nutritional Sciences, University of Kentucky College of Medicine, Lexington, Kentucky, USA; 2 Department of Bioengineering, University of Toledo College of Engineering, Toledo, Ohio, USA; 3 Markey Cancer Center, University of Kentucky, Lexington, Kentucky, USA; 4 Barnstable Brown Diabetes Center, University of Kentucky College of Medicine, Lexington, Kentucky, USA

**Keywords:** addiction, androgen receptor, cancer, estrogen receptor, glucocorticoid receptor, metabolism, mineralocorticoid receptor, nuclear receptors, PamGene, progesterone receptor

## Abstract

Steroid receptors are ligand-induced transcription factors that have broad functions among all living animal species, ranging from control of sex differences, body weight, stress responses, and many others. Their binding to coregulator proteins is regulated by corepressors and coactivators that interchange upon stimulation with a ligand. Coregulator proteins are an imperative and understudied aspect of steroid receptor signaling. Here, we discuss steroid receptor basics from protein domain structures that allow them to interact with coregulators and other proteins, their essential functions as transcription factors, and other elemental protein–protein interactions. We deliberate about the mechanisms that coregulators control in steroid receptor signaling, sex hormone signaling differences, sex hormone treatment in the opposite sex, and how these affect the coregulator and sex steroid receptor complexes. The steroid receptor-coregulator signaling mechanisms are essential built-in components of the mammalian DNA that mediate physiological and everyday functions. Targeting their crosstalk might be useful when imbalances lead to disease. We introduce novel technologies, such as the PamGene PamStation, which make investigating the heterogeneity of the steroid receptor–coregulator complexes and targeting their binding more feasible. This review provides an extensive understanding of steroid receptor-coregulator signaling and how these interactions are intrinsic to many physiological functions that may offer therapeutic advantages.

The human genome encodes 48 nuclear receptor proteins acting as ligand-induced transcription factors. Five nuclear receptors are in the steroid receptor class (Table 1), serving as signaling mechanisms for commonly produced steroids within the human body, such as estrogens, progesterone, testosterone, mineralocorticoids, and glucocorticoids. These hormones are essential in regulating numerous body systems, including metabolism, reproduction, immune, cardiovascular, and cognitive systems (1, 2). Glucocorticoids are among the most widely prescribed drugs today and are renowned for their potent anti-inflammatory and immunosuppressive effects (1). Endogenous glucocorticoids, such as cortisol, are steroid hormones released from the adrenal cortex as part of the hypothalamus–pituitary–adrenal axis that primarily signals through the glucocorticoid receptor (GR) (1, 3), leading to the transcriptional control of its target genes (1, 4).

**Table 1:** Steroid receptors expressed in the human genome

Steroid receptor	Nuclear receptor classification	Protein isoforms	Chromosome location	Endogenous ligand agonist(s)
Estrogen receptor	NR3A1 and NR3A2	ERα, ERβ	6q25.1-q25.2 (ERα), 14q23.2-q23.3 (ERβ)	Estradiol, estrone, estriol
Glucocorticoid receptor	NR3C1	GR⍺, GRβ	5q31.3	Cortisol
Mineralocorticoid receptor	NR3C2	MR	4q31.23	Aldosterone, cortisone, cortisol
Progesterone receptor	NR3C3	PR-A, PR-B	11q22.1	Progesterone
Androgen receptor	NR3C4	AR-A, AR-B	Xq12	Testosterone, dihydrotestosterone

In general, there are four states of nuclear receptor signaling: 1) ligand-induced transcriptional activation, 2) ligand-induced transcriptional repression, 3) ligand-independent transcriptional activation, and 4) ligand-independent transcriptional repression. These actions are essential to steroid receptor signaling function and their physiological responses (1).

Canonical signaling mechanisms of steroid receptors are regulated by proteins bound in the unstimulated, unliganded state, which changes upon a ligand binding to its ligand-binding domain (LBD) (1). An early example of GR-bound proteins is the immunophilin FK506-binding protein-51 (FKBP51) that interacts with GR when no ligand is bound (5), and once bound to glucocorticoids, FKBP52 binds the steroid receptor complex (6), replacing FKBP51 (7, 8). This ligand-induced protein swapping reorganizes the steroid receptor complexes, allowing GR to bind coregulator proteins and DNA to initiate gene transcription (6, 9, 10, 11). Most notably, coregulator proteins are coactivators or corepressors with a distinct function for each steroid receptor. The coregulator proteins do not have the same general tasks on all nuclear receptors, such as histone acetyltransferases and histone deacetylases (HDACs) have on gene function. Some coregulators are positive signaling mediators of the female sex hormone receptors while affecting male receptors differently, and we elaborate on these sex differences.

This review discusses the role of coregulators in controlling steroid receptor signaling mechanisms, sex steroid receptors, sex hormone differences among coregulators, sex hormone treatment in the opposite sex, and coregulators that have inverse functions across different nuclear receptors. We also discuss some coregulator proteins that may serve as targets for potential therapeutics and recent technological advancements, allowing for a broad study of coregulators interacting with a nuclear receptor.

## Steroid receptors

### Overview of nuclear receptor structure and function

**Figure 1:**
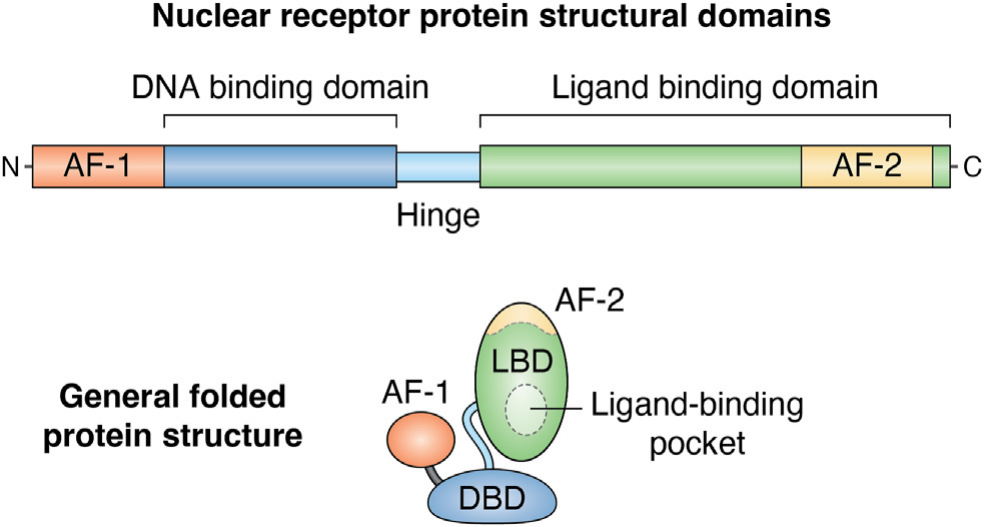
**The protein domain structures of nuclear receptors.** The protein domain structures of a nuclear receptor include the N-terminal domain, containing the activation function-1 (AF-1), DNA-binding domain (DBD), hinge region, ligand-binding domain (LBD), AF-2, and the C-terminal domain.

Structurally, nuclear receptors contain the following domains: N-terminal domain, DNA-binding domain (DBD), hinge region, LBD, and C-terminal domain (Fig. 1). The N-terminal domain contains the activation function-1 (AF-1) region, while AF-2 is localized in the LBD at the C terminus. The DBD binds to hormone response elements typically located in the gene's promoter, which are docking sequences targeted by a specific nuclear receptor for its regulatory gene function (12). The binding of coactivators or corepressors to the AF regions of nuclear receptors promotes the activation or suppression of gene transcription, respectively (13, 14). Nuclear receptors are typically bound to inhibitory proteins (FKBP51 for GR) or corepressor coregulator proteins when not bound to a ligand. Once the ligand binds, the inhibitory proteins are removed, switching from corepressors to coactivators. It should be noted that the actions of coactivators and corepressors are specific to each nuclear receptor, and their effects may vary among different nuclear receptors. Hence, there is no way to classify all coregulator proteins as coactivators or corepressors; it depends upon their specific actions on each nuclear receptor.

In contrast to ligand-induced transcriptional activation or repression, nuclear receptors also have ligand-independent transcriptional activation and repression. This activity has been observed to be frequent in some cancers and is dependent upon coregulator interaction. For example, in breast cancer, coactivator-associated arginine methyltransferase 1 acts as a coactivator for estrogen receptor (ER) α upon phosphorylation by PKA, allowing cAMP to activate ERα transcription (15).

### Steroid receptors and coregulator binding

**Figure 2:**
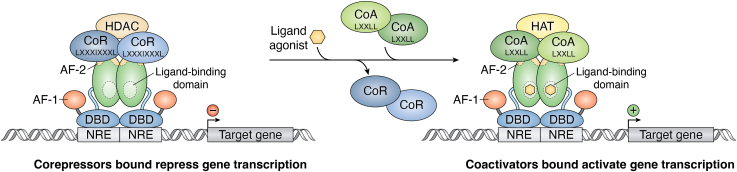
**Coregulator control of nuclear receptor–induced transcription.** Nuclear receptors dimerize and bind to gene promoters at nuclear receptor response elements (NREs). Corepressor coregulator proteins bind to the activation function-2 (AF-2) domain with the LXXIXXL motif and recruit histone deacetylases (HDACs). Ligand binding to the ligand-binding domain (LBD) within the AF-2 domain induces a change of corepressors to coactivators that bind *via* the LXXLL motif and recruit histone acetyltransferases (HATs). Coactivators binding to the nuclear receptor complex assist with transcriptional activation, allowing the target genes to be expressed.

The binding of a ligand causes conformational changes within the steroid receptor, allowing for coregulator interactions (1, 13). GR is a highly studied nuclear receptor and was the first in this class to have its gene cloned (16). Then, the mineralocorticoid receptor (MR) was cloned based on its hybridization to GR complementary DNA (cDNA) because of their similarities (17). Much has been investigated on GR’s coregulator interactions, which we discuss in this section to provide a general overview and later compare to the sex hormone receptors. Coregulators play a significant role in modulating the transcriptional activity of GR (1, 13). Coactivators and corepressors contain highly conserved motifs that bind to the AF-2 domain (Fig. 2) (1). Coregulators that bind to the AF-1 region do not have a sequence motif and bind independently of the ligand, unlike AF-2 domain binding, which is ligand-dependent (18).

Glucocorticoid treatment upregulates target gene expression by recruiting coactivator binding and inducing the disassociation of corepressors (1, 7), meaning that coactivator interaction with GR depends on ligand binding (1, 19). Glucocorticoids bind to GR and drive target gene expression by binding to glucocorticoid-response elements (GREs) within the DNA commonly found in the promoter region of a gene (20). The two main GR isoforms, GRα and GRβ, are derived from alternative splicing varying in the LBDs, with GRβ not having a glucocorticoid-binding function (1, 4, 21, 22, 23, 24, 25, 26). The activity and function of the GRα isoform are influenced by allosteric interactions, particularly in the AF-2 region of the LBD binding site, which affects ligand binding and coregulator interaction with the nuclear receptor that induces a conformation change in the protein (27). These allosteric modulations hinge on helix H12, a structural residue that bridges these two key sites. This bridge gains stability from the Trp600–Gln760 bond, highlighting its potential as a strategic target for modulating GR activity (28).

### Coactivators

Coactivators that bind to the AF-2 share a common amino acid motif of LXXLL-containing leucine, any two amino acids, and then two more leucine amino acids (Table 2). The leucine-rich sequence makes their structure hydrophobic, allowing them to interact with the hydrophobic groove of the nuclear receptor. The most commonly induced coactivator proteins have recently been renamed from their original nomenclature to nuclear receptor coactivator (NCOA) proteins. The NCOA proteins are a family of coactivators that bind, induce chromatin remodeling, and recruit the binding of additional coregulators and HATs (29, 30). When GR is bound to a ligand, it preferentially interacts with NCOA2, which acts as a scaffold, allowing other coactivators to bind, such as p300 (31). This interaction is unique because p300 is an HAT protein and contains two LXXLL domains in its N terminus. MR is bound by p300 at the LXXLL domain–containing amino acid sequence LSELL, but GR-bound p300 preferentially recruits other coactivators to the AF-1 domain rather than the LBD (32, 33).

**Table 2:** Coregulators known to interact with steroid receptors

Coregulator	GR (NR3C1)	MR (NR3C2)	PR (NR3C3)	AR (NR3C4)	ER⍺ (NR3A1)	ERβ (NR3A2)
ANDR	⚫️⚫️	⚫️	⚫️	⚫️⚫️	⚫️	
BL1S1	⚫️	⚫️	⚫️	⚫️	⚫️⚫️	⚫️
BRD8	⚫️	⚫️	⚫️	⚫️	⚫️	⚫️
CBP	⚫️⚫️	⚫️⚫️	⚫️⚫️⚫️⚫️⚫️	⚫️⚫️	⚫️⚫️⚫️	⚫️⚫️
CND1				⚫️	⚫️	
CENPR	⚫️	⚫️	⚫️	⚫️	⚫️	⚫️
CHD9	⚫️⚫️	⚫️⚫️	⚫️		⚫️	
CNOT1	⚫️⚫️⚫️⚫️	⚫️⚫️⚫️	⚫️⚫️		⚫️	
DDX5					⚫️	
DHX30	⚫️⚫️	⚫️			⚫️	
EP300	⚫️⚫️	⚫️⚫️⚫️	⚫️⚫️	⚫️⚫️	⚫️⚫️	⚫️⚫️
GELS	⚫️		⚫️	⚫️⚫️	⚫️	
GNAQ	⚫️		⚫️	⚫️		⚫️
HAIR	⚫️	⚫️	⚫️	⚫️	⚫️	⚫️
IKBB	⚫️	⚫️	⚫️⚫️⚫️	⚫️	⚫️⚫️	⚫️⚫️
ILK	⚫️		⚫️	⚫️⚫️	⚫️	⚫️
JHD2C	⚫️⚫️	⚫️	⚫️	⚫️⚫️	⚫️	⚫️⚫️
KIF11						
L3R2A			⚫️		⚫️	
LCOR	⚫️	⚫️	⚫️	⚫️⚫️	⚫️⚫️	⚫️
MAPE	⚫️		⚫️⚫️⚫️			
MED1	⚫️⚫️⚫️	⚫️	⚫️⚫️	⚫️⚫️	⚫️⚫️⚫️	⚫️⚫️⚫️
MEN1			⚫️	⚫️		
MGMT			⚫️	⚫️		
MLL2	⚫️⚫️	⚫️⚫️	⚫️	⚫️⚫️	⚫️⚫️	⚫️
MTA1S			⚫️	⚫️⚫️		
NCOA1	⚫️⚫️⚫️⚫️	⚫️⚫️⚫️⚫️	⚫️⚫️⚫️⚫️	⚫️⚫️⚫️⚫️	⚫️⚫️⚫️⚫️	⚫️⚫️⚫️⚫️
NCOA2	⚫️⚫️⚫️	⚫️⚫️	⚫️⚫️⚫️⚫️	⚫️⚫️	⚫️⚫️⚫️	⚫️⚫️⚫️
NCOA3	⚫️⚫️⚫️⚫️⚫️⚫️⚫️	⚫️⚫️⚫️⚫️	⚫️⚫️⚫️⚫️⚫️⚫️⚫️	⚫️⚫️⚫️⚫️⚫️	⚫️⚫️⚫️⚫️⚫️⚫️	⚫️⚫️⚫️⚫️⚫️⚫️⚫️
NCOA4	⚫️⚫️		⚫️⚫️	⚫️⚫️⚫️	⚫️	
NCOA6	⚫️⚫️⚫️	⚫️	⚫️⚫️	⚫️⚫️⚫️	⚫️⚫️	⚫️⚫️
NCOR1	⚫️⚫️⚫️	⚫️	⚫️⚫️⚫️⚫️	⚫️⚫️	⚫️⚫️	⚫️⚫️
NCOR2	⚫️	⚫️	⚫️⚫️⚫️	⚫️⚫️	⚫️	⚫️
NELFB	⚫️	⚫️	⚫️⚫️	⚫️⚫️		⚫️
NR0B1	⚫️⚫️⚫️⚫️	⚫️⚫️	⚫️⚫️⚫️⚫️	⚫️⚫️⚫️	⚫️⚫️⚫️⚫️	⚫️⚫️⚫️⚫️
NR0B2	⚫️⚫️⚫️⚫️	⚫️⚫️⚫️	⚫️⚫️⚫️⚫️⚫️	⚫️⚫️⚫️	⚫️⚫️⚫️⚫️	⚫️⚫️⚫️
NRBF2						
NRIP1	⚫️⚫️⚫️⚫️⚫️⚫️⚫️⚫️⚫️⚫️⚫️	⚫️⚫️⚫️⚫️⚫️⚫️⚫️⚫️⚫️	⚫️⚫️⚫️⚫️⚫️⚫️⚫️⚫️⚫️⚫️⚫️	⚫️⚫️⚫️⚫️⚫️⚫️⚫️⚫️⚫️⚫️	⚫️⚫️⚫️⚫️⚫️⚫️⚫️⚫️⚫️⚫️⚫️	⚫️⚫️⚫️⚫️⚫️⚫️⚫️⚫️⚫️⚫️
NSD1	⚫️	⚫️	⚫️	⚫️⚫️	⚫️⚫️	⚫️
PAK6	⚫️		⚫️	⚫️⚫️	⚫️	⚫️
PCAF	⚫️	⚫️⚫️	⚫️⚫️	⚫️		
PELP1	⚫️⚫️⚫️	⚫️	⚫️⚫️⚫️⚫️⚫️	⚫️⚫️	⚫️⚫️⚫️	⚫️⚫️⚫️
PIAS2	⚫️⚫️		⚫️⚫️	⚫️	⚫️	⚫️
PNRC1				⚫️		⚫️⚫️
PNRC2				⚫️	⚫️	⚫️
PPRC1	⚫️	⚫️⚫️	⚫️⚫️	⚫️	⚫️	⚫️
PR285	⚫️	⚫️	⚫️⚫️⚫️	⚫️⚫️	⚫️	⚫️
PRDM2			⚫️		⚫️	
PRGC1	⚫️⚫️⚫️⚫️	⚫️⚫️⚫️	⚫️⚫️⚫️	⚫️⚫️	⚫️⚫️⚫️⚫️	⚫️⚫️⚫️⚫️
PRGC2	⚫️	⚫️	⚫️	⚫️	⚫️⚫️	⚫️
PRGR			⚫️⚫️		⚫️	
PROX1	⚫️	⚫️⚫️	⚫️	⚫️	⚫️	⚫️
RAD9A	⚫️		⚫️	⚫️⚫️		
RBL2	⚫️					
TF65	⚫️		⚫️	⚫️	⚫️	
TGFI1	⚫️		⚫️	⚫️⚫️		
TIF1A	⚫️⚫️	⚫️⚫️	⚫️⚫️⚫️	⚫️	⚫️⚫️⚫️	⚫️⚫️
TIP60	⚫️⚫️	⚫️	⚫️	⚫️⚫️	⚫️⚫️	⚫️⚫️
TREF1	⚫️	⚫️⚫️	⚫️⚫️⚫️	⚫️	⚫️	⚫️
TRIP4				⚫️	⚫️	⚫️
TRAP	⚫️⚫️	⚫️⚫️	⚫️		⚫️⚫️⚫️	⚫️
TRXR1	⚫️	⚫️	⚫️	⚫️	⚫️⚫️	⚫️
UBE3A	⚫️⚫️	⚫️⚫️	⚫️⚫️	⚫️⚫️⚫️	⚫️⚫️⚫️	⚫️⚫️⚫️
WIPI1	⚫️	⚫️	⚫️⚫️	⚫️⚫️	⚫️⚫️	⚫️⚫️⚫️
ZNHI3	⚫️	⚫️	⚫️	⚫️	⚫️	⚫️⚫️
ZNT9			⚫️		⚫️	

The list of coregulators that have been shown to bind to steroid receptors. The data were adapted from Broekema *et al.* for the Nuclear Hormone Receptor (NHR) PamChip interactions with steroid receptors (76). The number of circles represents the number of LXXLL or LXXXIXXXL motifs within the coregulator protein that interacts with the steroid receptor.

AR, androgen receptor; ER, estrogen receptor; GR, glucocorticoid receptor; MR, mineralocorticoid receptor; NCOR, nuclear receptor corepressor; PR, progesterone receptor.

### Corepressors

Corepressors bind to the AF-2 domain with a distinct LXXI/HIXXXI/L motif containing leucine, any two amino acids, isoleucine or histidine, isoleucine, and three amino acids, and then a single isoleucine or leucine (34). Corepressors binding to nuclear receptors recruit HDACs, causing suppression of its target genes (1, 35). This is one way GR produces anti-inflammatory effects, such as its transcriptional repression of inflammatory mediators such as interleukin 6 (IL6). GR can also tether to NF-κB to prevent its signaling (36). Nuclear receptor corepressor (NCOR) binds to GR, recruiting transcriptional inhibitors such as HDAC3, decreasing the transcription of target genes (37). The NCOR class regulates nuclear receptor function and is critical for gene regulatory actions. These coregulators are vital proteins in the signaling mechanisms of glucocorticoids, suppressing the immune system and functioning as strong anti-inflammatory drugs (1).

## Sex hormone differences and steroid receptors

The ERα and ERβ isoforms, progesterone receptor (PR), and androgen receptor (AR) are involved in the differential sex development of organs such as breasts, ovaries, and testes and control overall reproduction through regulation by their specific sex hormones (Table 1). While much attention has been focused on the effect of estrogens in women and androgens in men, estrogen has an important role in men, and *vice versa*, androgen has an important role in women. AR has a preeminent role in developing prostate cancer, and possibly bladder cancer (38), and the use of specific estrogens has shown promise in treating the disease (39). One of the most common treatments for these cancers is hormone therapy. Tamoxifen is a competitive antagonist for ER and prevents the binding of coactivators that facilitate DNA binding and thereby inhibit estrogen’s genomic effects, reducing proliferation (40). About 30 to 40% of breast cancers have ER mutations, which contribute to hormone therapy resistance (41). Most mutations occur within the ER LBD, interfering with its ability to bind estrogen and to be inhibited by antiestrogens, contributing to hormone therapy resistance and constitutive activity (42). These mutated ERs' activity depends on their interactions with coregulators (41), and we discuss this aspect further below. For prostate cancer, antiandrogen drugs such as bicalutamide work similarly to tamoxifen-ER, and it competitively bind to AR to prevent testosterone activation (43) in prostate cancer. The biggest challenge that hormone therapy faces is resistance, which occurs in most patients, underscoring the need for improved treatments. The benefits and negatives of using sex hormones as a therapeutic in the opposite sex are discussed further below.

### Interaction of ER and AR

**Figure 3:**
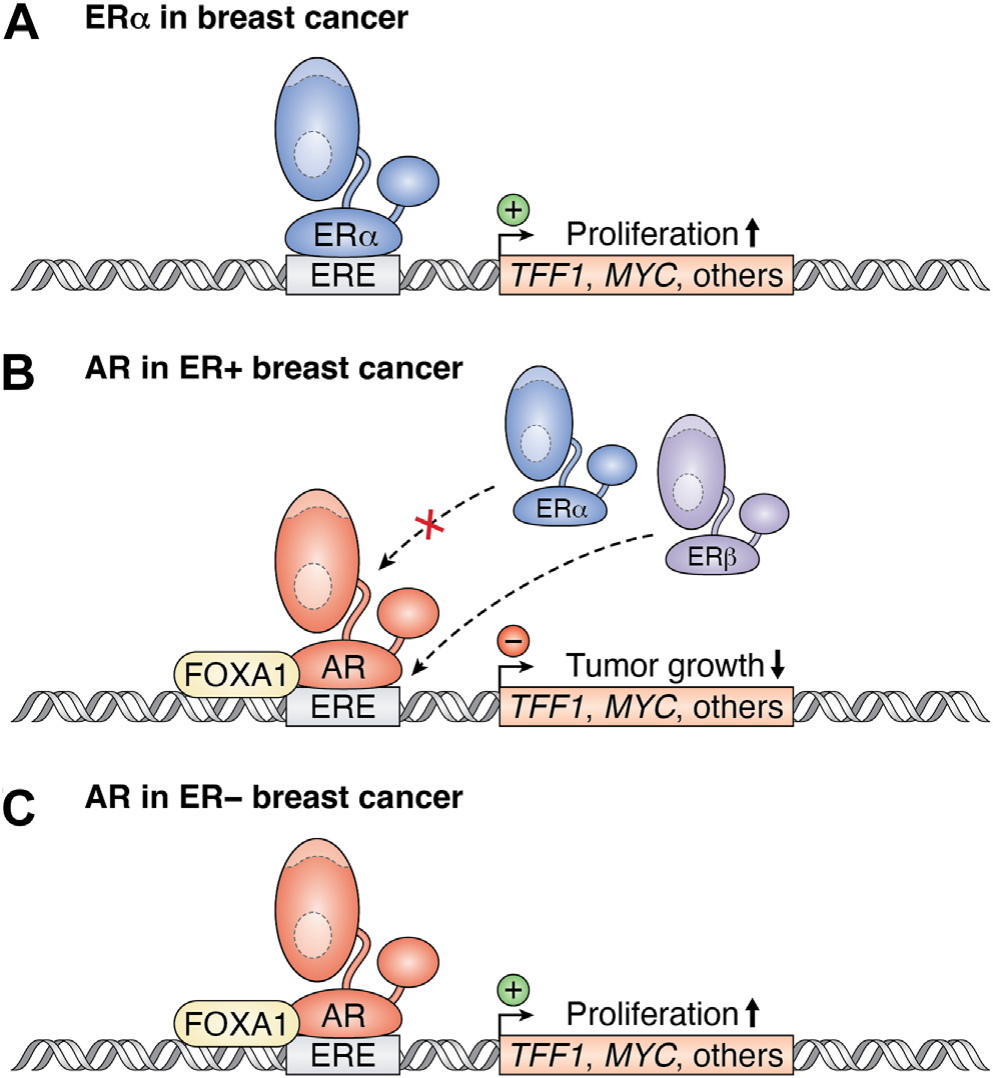
**ER and AR signaling crosstalk.***A*, in breast cancer, ERα binds to the estrogen response element (ERE), increasing the transcription of proliferation signals, such as *TFF1* and *MYC*. *B*, AR has a bidirectional role in breast cancer, depending on the presence of ERα. In ER-positive breast cancer, AR and FOXA1 bind to the ERE, blocking ERα binding and transcription and preventing tumor growth. ERβ can also bind and inhibit the transcription of proliferation signals. *C*, in ER-negative breast cancer, AR and FOXA1 bind to the ERE and increase transcription of genes to promote proliferation and tumor growth. AR, androgen receptor; ER, estrogen receptor; FOXA1, Forkhead box A1.

AR may inhibit ER’s transcriptional effects by binding to estrogen response elements (EREs) and blocking it from binding (Fig. 3) (44). Similarly, ER has an inhibitory function against androgen-induced AR transactivation (45). ERα and AR have been shown to colocalize in brain cells and often translocate simultaneously, forming heterodimers (45). A study using fluorescently labeled AR and ERα revealed that AR subcellular localization changed in response to estradiol, ERα localization responded to testosterone, and estradiol-bound ERα increased AR mobility (45).

In breast cancer cells, synthetic androgens activate AR, upregulating ERβ expression and inhibiting growth (46). Some evidence shows that AR and ER work synergistically to manifest breast cancer by upregulating kallikreins, known markers for breast cancer (47). The ER/AR interaction is also relevant in gender dysphoria (48). However, little is known about their roles and the interactions between ER, AR, and coregulators. Given that sex hormone steroid receptors are involved in many bodily processes, more studies are needed to better understand the coregulators and their functions.

### Androgens and breast cancer

Most breast cancers express AR, regardless of whether they express ER (49, 50). The role of AR in breast cancer depends on whether ER is present and its activity (Fig. 3) (51). When ER is expressed and active, AR blocks its activity, lowering the tumor cell growth (51). In a cohort of over 1100 breast cancer patients, AR expression and nuclear localization were associated with lower tumor size and grade, leading to a better prognosis in ER-positive tumors (52). In breast cancer, elevated ERα expression with low AR increased the risk of cancer-related deaths by 4.6-fold (44). Studies in ER-positive and apocrine breast cancer cell lines identified that AR inhibits ER-induced transcription and proliferation effects by binding to EREs to prevent the binding of ER (44). Robinson *et al.* found that the binding of AR to the EREs in breast cancer was dependent on the presence of Forkhead box A1 (50). In the absence of ER, AR activates tumor cell growth (51). In the human ER-breast cancer cell line MDA-MB-453, 5α-5-dihydrotestosterone (DHT) significantly increased cell growth, which was attenuated by cotreating with the AR antagonist bicalutamide (53).

In ER-negative (ER−) breast cancer, the activation of AR leads to the upregulation of Wnt and epidermal growth factor 2 (HER2) protumorigenic signaling pathway by increasing the transcription of Wnt family member 7B and epidermal growth factor 3 (HER3) (53). In HER2+ ER− breast cancer cells, the inhibition of AR decreased cell growth and caused a reduction in HER2 phosphorylation and the ERK/AKT growth pathway (54). The protective role of AR in ER-positive breast cancer makes it a potential target for regulating tumor growth. However, its opposing roles in ER-negative breast cancer limit its potential uses.

### Androgen excess in women and insulin resistance

Androgen excess is an endocrine disorder that affects 20% of women and heightens the risk for insulin resistance and metabolic dysfunction (55). Conversely, androgen deficiency puts males at risk of metabolic dysfunction (56). Liver-specific AR KO female mice were prevented from DHT-induced insulin resistance but did not affect healthy levels of circulating androgens (57). Furthermore, neuronal AR KO mice develop insulin resistance and increased fat mass in mice (58). Without AR in the hypothalamus, the ability of insulin to phosphorylate AKT was impaired, and the AR suppression of NF-kB–induced inflammation was lost (58).

### Estrogen influence on addiction

Clinical sex differences in the trajectories of substance use disorders, including nicotine, opioids, and psychostimulants (59, 60, 61, 62), indicate that women are more susceptible to addiction than men (63, 64, 65). These have led to mechanistic preclinical evaluations of how estrogens impact drug use, withdrawal, and relapse (66, 67, 68). Estrogens modulate the neurophysiology of GABAergic medium spiny neurons within the striatum (69, 70, 71). These cells express ERs, which colocalize with metabotropic glutamate receptors (mGluRs) and are critical in driving addiction-related behaviors (72, 73).

Estrogen activates ERα and ERβ, but the specific roles of each receptor in substance use disorders are unknown. The primary function of ER, like GR, is activated when it binds to its ligand, stimulating transcriptional control of numerous genes *via* binding to EREs (74). However, the tissue distribution of ERα and ERβ are diverse, and the responsiveness is regulated differently based on the tissue. For instance, ERα upregulates the UDP-glucuronosyltransferase 1A1 enzyme in the liver, but ERβ activation suppresses it (75). The inverse functionality of ERα and ERβ may be from differential coregulator binding as Broekema *et al.* found that 17β-estradiol treatments using purified protein showed that ERα and ERβ were bound to many of the same coregulators (NCOA1, NCOA2, NCOA3, NCOR1, NRIP1, PELP1, and several others) (76). However, they found that some coregulators had selective binding to ERα or ERβ; ERα bound IKBB, TRRAP, TIF1A, and CBP, while ERβ selected GNAQ, NELFB, and PNRC1 (76). How the coregulators function to control ERα or ERβ in the brain during addiction is unknown, and more studies are needed.

The ER isoforms also have functions independent of binding to gene promoters *via* protein–protein interaction with activator protein-1 transcription factor (77). Ligand-induced activation of ERα stimulates G protein–coupled receptors to signal to the nucleus through mitogen-activated protein kinase signaling pathways (78). Specifically, activation of ERα activates mGluR1 (Gq) or mGluR2/3 (Gi/o), leading to either activation or inhibition of cAMP response element–binding protein (CREB) phosphorylation (73). When mGluR1 is stimulated, phospholipase C (79) activates PKC and inositol trisphosphate pathways, activating mitogen-activated protein kinase (80) and inducing translocation of p90 ribosomal protein S6 kinase into the nucleus to induce CREB phosphorylation and thereby influence gene transcription (81). Estrogen bidirectionally influences CREB phosphorylation *via* mGluR2/3, whereby ERα activation inhibits adenylyl cyclase and cAMP/PKA, decreasing L-type calcium channel–dependent CREB phosphorylation (73, 82). Activation of CREB within the nucleus accumbens core has been demonstrated to reduce the sensitivity to reinforcing effects of addictive drugs, therefore increasing tolerance and self-administration (83, 84, 85, 86). Thus, estrogen-induced CREB inhibition may be a critical mechanism by which this steroid hormone acts to enhance addiction-related behavior.

Few studies have brought estrogen intracellular signaling mechanisms into focus within preclinical addiction models. Most of the studies conducted were with cocaine by the Mermelstein group (87, 88, 89), and we have evaluated estrogen modulation of nicotine addiction and accumbens neurobiology in females (71, 90). These findings provide an important framework upon which future studies can define specific estrogen-dependent mechanisms within the brain reward pathway underlying female-specific enhancement of addiction vulnerability.

### Estrogens and prostate cancer

Prostate cancer is attributed to abnormal activity of AR. However, a study by Lucas *et al.* demonstrated that in rats, estradiol activates ERα and ERβ to stimulate the proliferation of immature Sertoli cells, therefore playing a positive role in spermatogenesis (91). Mice with a global ERα KO were found to be infertile, had lower sperm count, and the sperm they were able to produce were dysfunctional (92). Studies in humans have shown the opposite, where infertile men had higher serum ERα and estradiol levels than fertile men (93). The coregulator PELP1 (proline, glutamic acid, and leucine-rich protein-1) contains a commonly shared LXXLL domain within amino acids 20 to 42 (GTGGLSAVSSGPRLRLLLLESVS) that binds both ER isoforms and AR (76). Interestingly, PELP1 has been implicated in prostate cancer due to its role in enhancing AR transcription (94). However, the coregulator modulation of ERs in prostate cancer remains unknown.

ERβ activation has been demonstrated to inhibit prostate cancer progression. Guerini *et al.* found that a derivate of androgen, 3β-adiol, activates ERβ in the prostate and inhibits migration (95). ERβ is highly expressed in the normal prostate; however, one study found that in over 75% of prostate cancers, ERβ was found not to be expressed (96). In an *in vitro* model of prostate cancer, it was shown that estrogen and selective ER agonists affected prostate cancer growth but only in AR-positive cell lines (44). In another cell line, 22Rv1, ER antagonist tamoxifen had a significant antiproliferative effect independent of ERs (39). While it is widely hypothesized that ERα activity promotes cancer growth and ERβ inhibits it, there may be an opposite effect on the tumor immune microenvironment (97).

### ERs and liver disease

Estrogens generally protect against hepatocellular carcinoma (HCC) by activating the transcription of several protective genes, such as *MTA1*, *TP53*, and *PTPRO* (98). The ratio of ERα to ERβ is important in prostate and breast cancers, and recent evidence suggests that it is also important in hepatic and colon cancers. Lower expression of ERβ is highly associated with cancer progression (96, 99, 100, 101).

**Figure 4:**
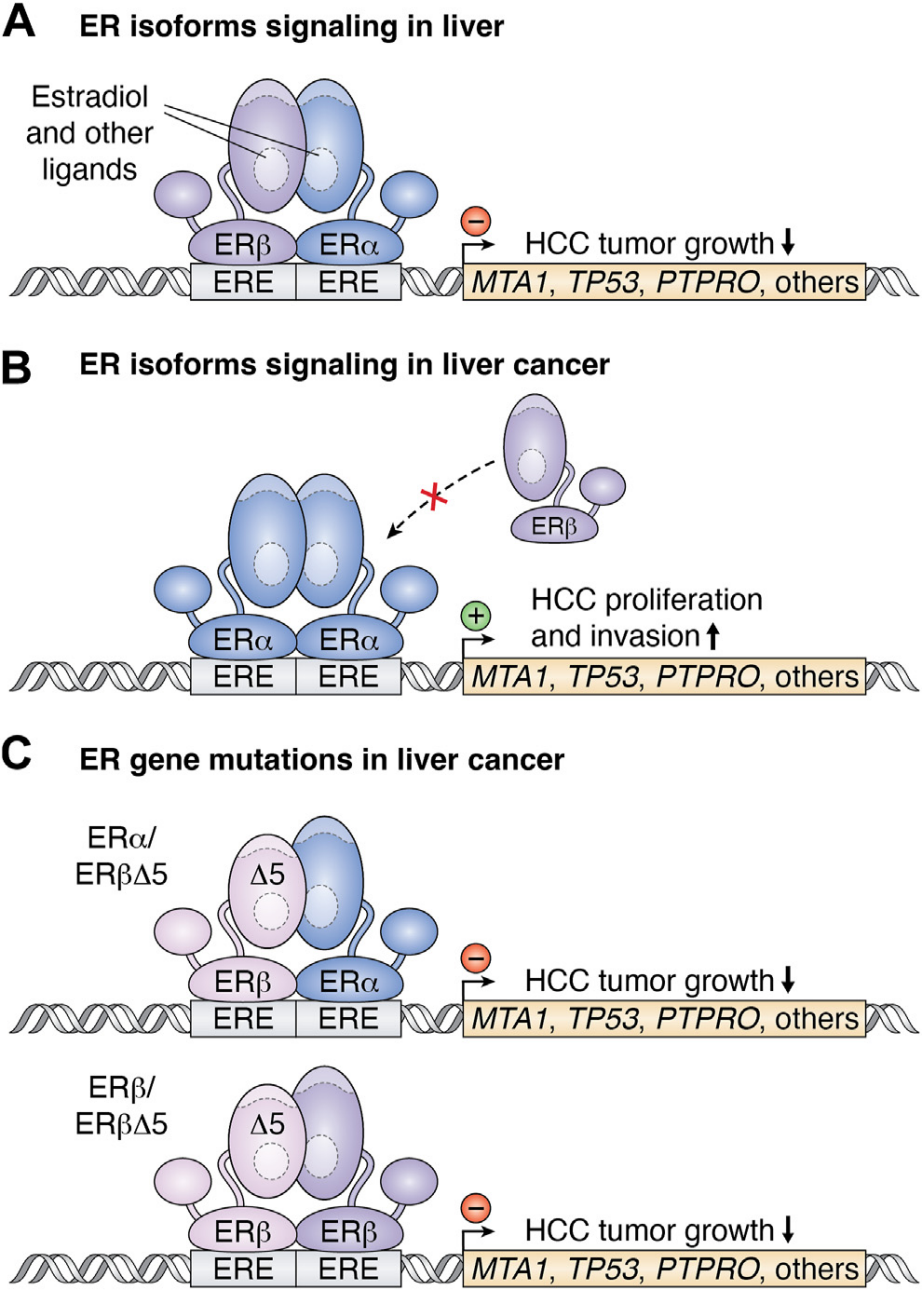
**Estrogen receptor isoforms and their variants in hepatocellular carcinoma.***A*, normally, in tissues where ERα and ERβ are coexpressed, they dimerize to control gene function. Upon binding to the ERE, a complex with ERβ will inhibit the transcription of genes that ERα induces to increase HCC tumor growth, such as *MTA1*, *TP53*, and *PTPRO*. *B*, ERα with low levels of ERβ is pathogenic, and two ERα molecules homodimerize and increase HCC proliferation and invasion. *C*, ERβΔ5 is a splice variant that lacks exon 5 but still dimerizes with ERα or ERβ to prevent HCC growth. MTA1, metastasis-associated protein 1; ER, estrogen receptor; HCC, hepatocellular carcinoma.

A common ER splice variant that lacks exon 5 (ERαΔ5 and ERβΔ5) results in the loss of its functional LBD (Fig. 4) (102). ERαΔ5 expression was more prevalent in HCC patients, and ERβΔ5 had no difference (99). ERαΔ5 is associated with increased aggressiveness and shorter doubling time of the tumor and was identified as the strongest prognostic factor affecting survival in patients with HCC (103). This finding was also made by Iavarone *et al.*, who determined that male and female patients lacking the coexpression of ERα and ERβ had a higher frequency of HCC (99). The coexpression of ERα and ERβ was more prevalent in chronic liver disease compared to HCC, and elevated expression of ERα or splice variants, as well as lower intact ERβ, was found more often in those with severe liver disease and HCC (99). The dimerization of ER isoforms depends on the LBD and DBD, and deletion of the LBD impairs the ability to dimerize (104). Their coexpression results in differences in the homo and hetero dimers of ER isoforms and, therefore, changes their function and likely the coregulator binding within their steroid receptor complexes.

An area that needs to be explored further is the role of the ER isoform splice variants in liver disease, as the ERα/ERβΔ5 and ERβ/ERβΔ5 dimers form with the intact WT ER isoforms (Fig. 4) (105). ERβΔ5 has a dominant negative effect on ERα and ERβ because it significantly decreases transactivation and potentially generates transcriptionally inactive dimers with reduced transcriptional capacity (105). Despite evidence showing that ERβΔ5 is associated with improved liver disease status and that it can inhibit the activity of ERα, there have been no studies directly examining the role of ERβΔ5 on HCC. The antitumorigenic impacts of estrogen in HCC have been partially attributed to ER suppression of IL-1a and IL-6 transcription (106). More studies are needed on the ER isoforms and their coregulator involvement in HCC.

## Coregulators of steroid receptors as drug targets

Coregulators have long been of interest as potential targeting therapeutics for diseases such as cancer, Alzheimer’s, insulin-resistant diabetes, and obesity (107). The O’Malley group reported that 101 of 285 known coregulators (Table 2 contains coregulators interacting with steroid receptors) are overexpressed or underexpressed in cancer (107, 108). Not only is their altered expression associated with carcinogenesis but coregulators can also disrupt the efficacy of therapeutics that target nuclear receptors for cancer treatment. Shang *et al.* demonstrated that tamoxifen treatment, a selective estrogen receptor modulator, in endometrial cells mimicked the effects of estrogen on the recruitment of coactivators NCOA1, AIB1, and CBP to gene promoters targeted by the activation of ER (109). This effect was not observed in mammary cells, where tamoxifen treatment induced corepressors NCOR and SMRT to associate with ER (109). These concepts highlight a largely unexplored area of coregulator biology to be leveraged for drug development.

### Therapeutic disruption of PELP1 binding

**Figure 5:**
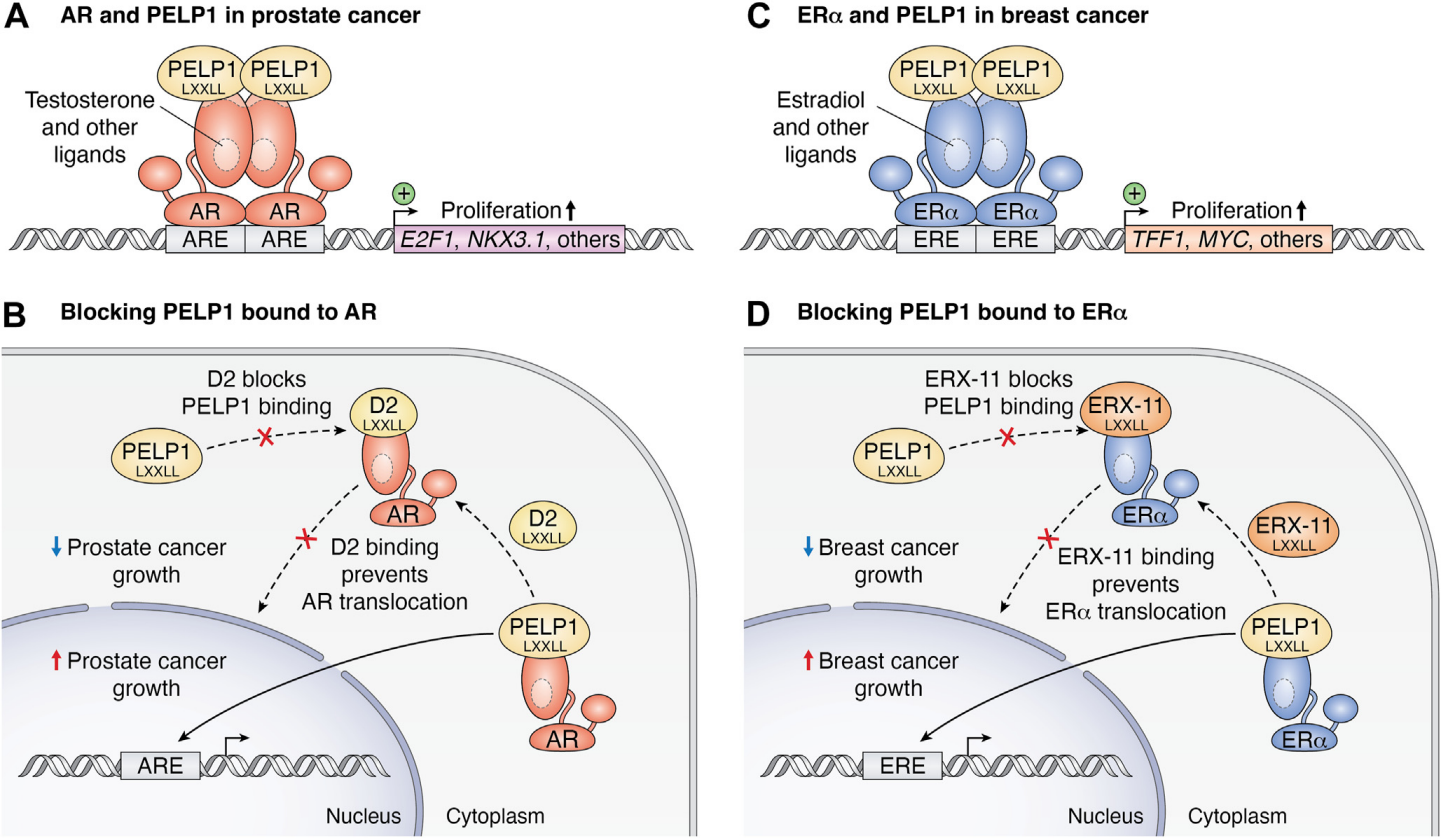
**Coregulator PELP1 is a druggable target in prostate and breast cancer.***A*, in prostate cancer, testosterone binds to the LBD in the AF-2 region of AR. PELP1 binds to the AF-2 region with its conserved coactivator LXXLL sequence. AR binds to the androgen response element (ARE) of gene promoters such as *E2F1* and *NKX3.1*, controlling prostate cell proliferation. *B*, the peptidomimetic D2 binds with an LXXLL sequence to block the binding of coregulators such as PELP1 to prevent translocation of AR and, therefore, prevent AR-induced prostate cancer growth. *C*, PELP1 also acts as a coactivator and binds to ERα, causing transcription of genes that promote proliferation in breast cancer cells, such as *TFF1*, *MYC*, and others. *D*, ERX-11 is a peptidomimetic that binds where the PELP1 LXXLL binds with the AF-2, displacing it from regulating ERα-induced proliferation pathways. ERX-11 binds to ERα and prevents binding to the ERE, inhibiting breast cancer growth. AR, androgen receptor; ER, estrogen receptor; ERX, ER coregulator-binding modulator; FOXA1, Forkhead box A1; LBD, ligand-binding domain.

PELP1 is a coregulator for several nuclear receptors that often acts as a scaffold and is a potential therapeutic target. PELP1 has 10 LXXLL domains that allow for interaction with many different nuclear receptors (110). It is a coactivator for ERα and ERβ and interacts with other steroid receptors such as AR, GR, PR, and MR (Table 2). PELP1 has been implicated in a multitude of cancers. Its deregulation has been demonstrated in endometrial, ovarian, breast, and prostate cancers (94, 110, 111, 112). Ravindranathan *et al.* used peptidomimetics to target the conserved α-helix in the LXXLL motif to inhibit PELP1 binding to the AF-2 region of AR to serve as a prostate cancer therapeutic (Fig. 5, *A* and *B*) (113). They found that their peptidomimetic, named D2, was able to specifically disrupt the binding of PELP1 to AR with an IC_50_ of 40 nM, preventing AR translocation, resulting in decreased growth of cancer cells *in vitro*, *in vivo*, and *ex vivo* (113). The authors concluded that targeting the PELP1–AR interaction disrupts AR translocation into the nucleus, inhibiting its target gene regulatory actions.

PELP1 binds to both ER isoforms (Table 2) (76) and may be useful for targeting ERα in breast cancer as it was shown to facilitate target gene regulation (114). Because many patients become resistant to selective estrogen receptor modulators, 60% of women have either intrinsic or acquired resistance (115); this has driven some research groups to focus on targeting the ER coregulators directly. Despite predictions that the D2 peptidomimetic would likely block the coregulator interactions with ER, Raj *et al.* determined this was not the case, as D2 did not block the interaction between PELP1 and ERα (116). However, adding a functional group to D2 created an ER coregulator-binding modulator (ERXs). ERX-11 effectively binds the AF-2 domain with an IC_50_ of 250-500 nM and inhibits ER-induced proliferation (Fig. 5, *C* and *D*) (116). Coimmunoprecipitation (Co-IP) assays revealed that ERX-11 disrupts the complexes formed by PELP1 and ERα in MCF-7 cells. ERX-11 increased apoptosis and decreased xenograft breast cancer tumor volume (116). Distinct from tamoxifen, ERX-11 was still effective in therapy-resistant models, suggesting that it could be used as a monotherapy or in combination with tamoxifen.

### Nuclear receptor coactivators and cancer

The NCOA coregulators have been considered a potential target for cancer therapeutics. NCOA1 and NCOA3 are overexpressed in human breast cancer, and their inhibition or deletion has attenuated cancer progression in various models of multiple types of cancer (117, 118, 119). Qin *et al.* modified SI-2, the previously identified NCOA small molecular inhibitor, to improve its pharmacokinetics and therapeutic potential, creating SI-10 and SI-12 (120). They found that these compounds had a longer half-life, reduced NCOA3 levels, decreased proliferation and migration in breast cancer cells, and attenuated tumor growth in mice (120). NCOA3 has three LXXLL domains and is required for the transcriptional activity of ER and AR, but it has been shown to bind to all of the steroid receptors (Table 2). NCOA3 interacts with ERα and initiates a cascade by recruiting p300 and coactivator-associated arginine methyltransferase 1, resulting in proliferation (121, 122). NCOA2 is another coregulator that may be involved in prostate cancer. Knockdown of NCOA2 in a prostate cancer cell line cultured with DHT and the antiandrogen bicalutamide increased proliferation (123), suggesting that NCOA2 may inhibit prostate cancer growth.

### Metastasis-associated protein 1

Metastasis-associated protein 1 (MTA1) is a dual coregulatory protein overexpressed in various cancers, including castration-resistant prostate cancer. AR is the most critical transcription factor in prostate cancer, but other dysregulated pathways also exist, such as the MTA1/PTEN/AKT pathway. One group targeted this pathway by treating mice with gnetin C, a melinjo plant compound and inhibitor of MTA1 (124). MTA1 does not directly act as a coregulator for AR but interacts with other DNA-binding transcription factors that regulate its activity. Campanelli *et al.* demonstrated that gnetin C treatment in castration-resistant prostate cancer cells sensitized them to the AR antagonist enzalutamide treatment (125). It was concluded that this was mediated through AR-MTA1–mediated pathways, but the mechanisms are still unknown.

Altogether, these findings indicate that targeting coregulators may be a more reliable and consistent strategy to treat hormonally driven cancers such as prostate cancer or breast cancer, in which resistance to therapeutics is a frequent occurrence.

## Technology for studying coregulators

### Basic steroid receptor-coregulator binding techniques

**Figure 6:**
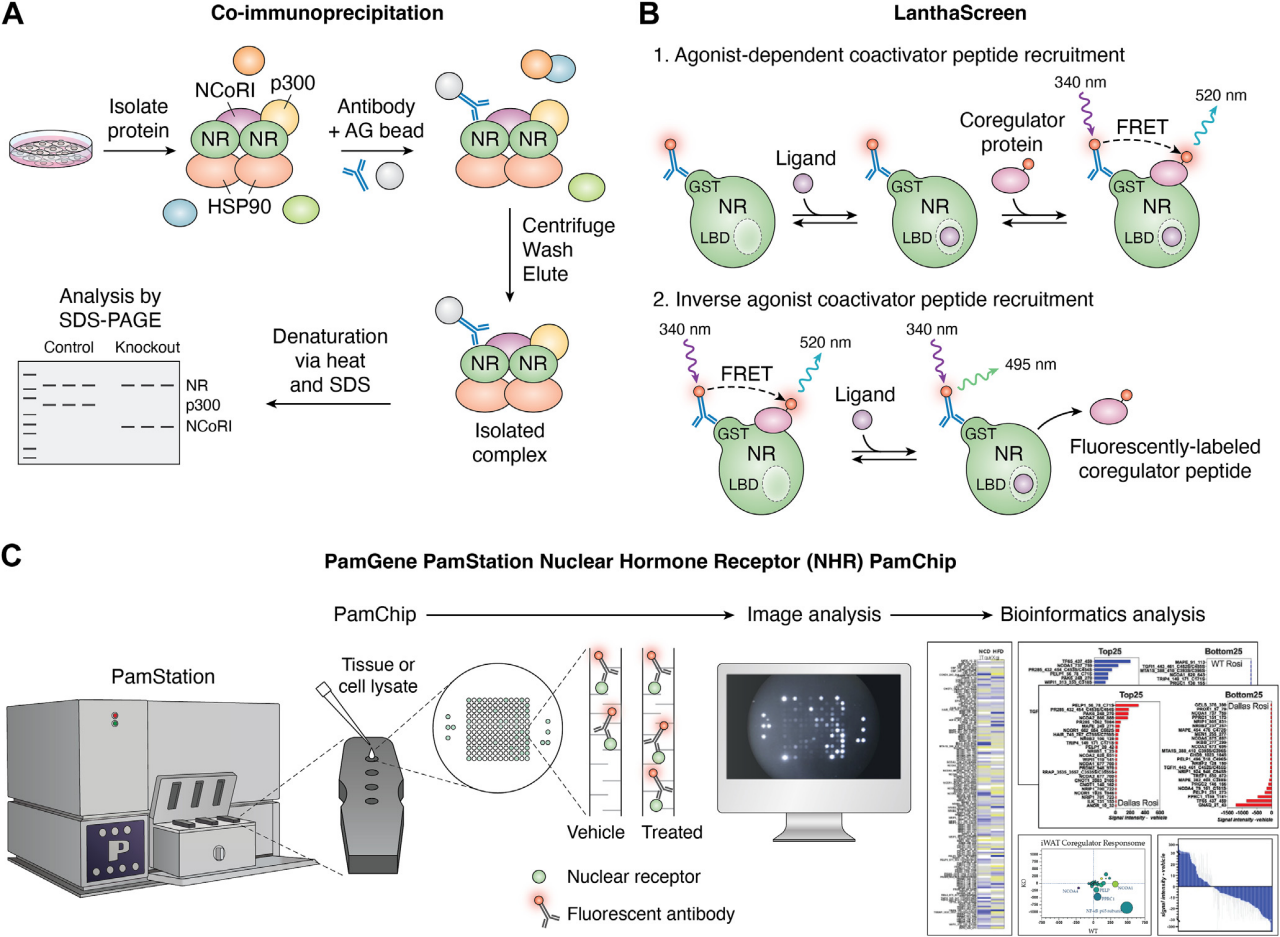
**Techniques to assess coregulator binding of nuclear receptors.***A*, the Co-IP technique first involves immunoprecipitating using a specific antibody against a particular protein. Once the protein of interest is immunoprecipitated and isolated from other nonspecific proteins, other proteins bound to a nuclear receptor can be assessed through Western blotting. A limitation of the Co-IP procedure is that they only analyze one coregulator interaction with a nuclear receptor. *B*, the LanthaScreen assay. The coregulator peptide is labeled with a terbium donor fluorophore, such as fluorescein. When the nuclear receptor is excited at 340 nm, the terbium will emit a 495 nm signal in response. When the coregulator peptide binds, the donor and acceptor fluorophore pair together, a 520 nm signal will be produced due to TR-FRET excitation. *C*, the NHR PamChip has 155 coregulator peptide motifs containing their specific LXXLL or LXXXIXXXL domains immobilized on a porous membrane for interaction and identification (142). A fluorescently labeled antibody identifies the nuclear receptor to indicate each coregulator’s location in the NHR PamChip array. The samples are prepared using proprietary buffers and the tissue or cell lysates or purified nuclear receptor protein is added to the NHR PamChip and then run for 120 cycles in the PamGene PamStation instrument, which moves the solutions through the membranes while taking images each cycle. These images are used for quantification of nuclear receptor–coregulator interaction. These images are then reviewed for quality control before data analysis. Data analysis steps for the NHR PamChip assay include a time-course–dependent equation that calculates the fluorescent signal intensity over the 120 cycles at five different time points. Co-IP, coimmunoprecipitation; NHR, nuclear hormone receptor; TR-FRET, time-resolved FRET.

There are limited experimental procedures to assess coregulator interaction with a nuclear receptor. Co-IP and LanthaScreen coregulator binding assays (Fig. 6) have been the most used research techniques for studying coregulator-nuclear receptor binding. Using classical Co-IP and immunoblotting techniques in the U2OS-rGR human osteosarcoma cell line, Dobrovolna *et al.* identified that the NCOA2 coregulator (the authors used the previous naming as GR-interacting protein 1), in addition to functioning as a general coactivator for GR, also selectively works to repress the activity of certain GR-mediated signaling pathways (126). Other groups have also identified the duplicity of coregulators in their ability to increase and suppress the transcriptional activity of a nuclear receptor using a combination of Co-IP and transcriptional reporter assays.

To assess the interaction of corepressor NCOR1 with GR, Wang *et al.* used a two-hybrid luciferase reporter assay in CV-1 cells (127). They showed a significant increase in the luciferase signal when GR was bound by the antagonist RU486 but not dexamethasone. The authors introduced mutations in the NCOR1 LXXXIXXXL motif to determine that NCOR1 interaction with GR is required for antagonistic and agonistic actions of GR using a similar dual-luciferase reporter assay (127). Their findings suggested that NCOR1 may play opposing roles in regulating GR when different chemicals are bound. The authors confirmed by Co-IP that NCOR1 is associated with GR when bound by RU486 and dexamethasone, a GR antagonist and agonist, respectively (127). They also found that GR is competitively bound to NCOA2 (the authors referred to the older naming of TIF2), which actively inhibits NCOR1 binding in both agonistic and antagonistic activation of GR (127). It is important to note that Co-IP assays are limited to one-to-one protein interactions and cannot assess the magnitude or how multiple coregulators work together to regulate the activity of nuclear receptors. These indicate a need to better understand the molecular characteristics of coregulator interaction as a complex with nuclear receptors.

### Single coregulator fluorescence-based binding assays

The LanthaScreen technology utilizes a time-resolved-FRET–based approach with a fluorophore biomolecular interaction to detect two nearby molecules of interest (Fig. 6). The energy transfer between the two fluorophores causes a dual emission effect, quantified as a ratio of acceptor to donor fluorophore intensity, which is utilized to determine the relative distance between two proteins of interest (128). The LanthaScreen contains three components: 1) a glutathione S-transferase (GST)-fused nuclear receptor LBD, 2) a single coregulator peptide labeled with fluorescein (terbium donor fluorophore), and 3) a specific ligand. The nuclear receptor LBD fused with GST allows an anti-GST terbium-labeled antibody to label the nuclear receptor (129). The terbium and fluorescein act as a fluorophore pair with detectable emissions that signal the relative distance between the nuclear receptor and coregulator.

The LanthaScreen technology has been utilized to determine nuclear receptor indirect and direct ligands for the constitutive androstane receptor (CAR) and coregulator peroxisome proliferator-activated receptor-gamma coactivator -1 alpha (128). They determined that agonist-dependent coactivator recruitment to CAR correlated with the potency of three different CAR ligands, including clotrimazole, PK11195, and androstenol (128). Using inverse-agonist coactivator peptide displacement, they identified that clotrimazole acts as an antagonist and inverse agonist of the CAR LBD, noting that peroxisome proliferator-activated receptor-gamma coactivator -1 alpha is displaced more robustly when CAR is bound by clotrimazole (128). It is important to note that each LanthaScreen assay must be optimized specifically for a particular nuclear receptor and coregulator peptide pair, and these kits and procedures do not currently exist for all nuclear receptor-coregulator–specific interactions.

### Multiple coregulator-binding analysis using PamGene instrumentation

i. Overview of the technology

The PamGene PamStation nuclear hormone receptor (NHR) PamChip, which utilizes the MARCoNI (microarray assay for real-time analysis of coregulator–nuclear receptor interaction) technology, quantifies the interaction of 155 coregulator protein motifs with a nuclear receptor (Fig. 6). This allows for studying numerous coregulator binding and dissociation from nuclear receptor complexes. The value of this technology is that 155 coregulator interactions are being assayed simultaneously, providing the magnitude of the coregulator binding (see Table 3, which contains studies on nuclear receptors using the PamGene NHR Technology). These aspects are essential for investigating ligand-specific gene pathways likely driven by coregulator complexes.

ii. Steroid receptors and NHR PamChip technology

**Table 3:** Nuclear receptor studies using the PamGene NHR technology

First author	Summary	Nuclear receptor	PMID
Monczor	They identified distinct coregulator profiles for three different GR ligands, including dexamethasone, RU486, and cyproterone. The GR-dexamethasone coregulator complex showed stronger associations than RU486 or cyproterone. Targeting of the coregulator NR0B1 with siRNA showed a difference in transcriptional response for only one GR target gene (SLP1) while maintaining the expression of other target genes.	GR	30930776
Aarts	This group screened 25 compounds, including ER agonists, antagonists, and endocrine disruptors, to determine differences in coregulator binding. They found that many endocrine disruptors had inverse coregulator binding tendencies when compared to ER agonists, such as NCOA1, which may be related to negative side effects related to phthalate exposure.	ERα	23383871
Houtman	This group illustrated the importance of the phosphorylation status of ERα for coregulator binding. They found that ERα serine 305 phosphorylation decreased interactions with NCOA1 when ERα was activated with 17-β-estradiol. Conversely, they found that ERα activation with tamoxifen increased NCOA1 binding despite the serine 305 phosphorylation status of ERα.	ERα	22319200
Perera	Using purified AR protein, this group examined the coregulator profile of endocrine disruptors that have been hypothesized to bind AR. They compared the binding profiles of BPA, BPAF, and BPS against AR agonists R1881 and CPA, noting that BPA and BPAF shared very similar coregulator profiles to that of CPA. Key findings of this study are that BPA, BPAF, and BPS share similar coregulators to known AR agonists that seem to both enhance and repress transcription. However, their molecular docking studies suggest that these endocrine disruptors may bind with higher affinity to AR, partially explaining the variety of coregulator binding patterns seen in their study.	AR	28751236
Koppen	This group identified that PPARγ has inverse binding affinities with NCOR1 and TRIP3 when activated by rosiglitazone (PPARγ agonist) but not GW9662 (antagonist). They show with the NHR technology how coregulators contribute to the activation or repression of transcription through ligand-specific interactions. This data led to the identification of TRIP3 as a vital regulator of adipocyte differentiation.	PPARγ	19596656
Gordon	This group uses purified PPARα LBD protein to show that WY-14,643 exhibited the strongest coregulator binding tendencies when compared to bilirubin and fenofibrate. When full-length PPARα protein was used, they found that bilirubin displayed the strongest coregulator binding, with WY-14,643 having the weakest interactions. Using adipose tissue lysate of mice treated with bilirubin nanoparticles, they found that NCOA1, NCOA2, and NCOA3 had increased interactions with PPARα, and several NRIP motifs had decreased interactions. These data indicated that bilirubin is likely activating PPARα and repressing PPARγ to elicit some of the fat-burning effects of bilirubin treatment.	PPARα	32404366

The list of NHR PamChip uses on steroid receptors and a summary of their findings.

AR, androgen receptor; BPA, bisphenol A; BPS, bisphenol S; BPAF, bisphenol AF; CPA, cyproterone acetate; ER, estrogen receptor; GR, glucocorticoid receptor; LBD, ligand-binding domain; NCOA, nuclear receptor coactivator; NHR, nuclear hormone receptor; TRIP3, thyroid hormone receptor-interacting protein 3.

Studies have been conducted on GR using the NHR PamChip technique to determine ligand-specific coregulator binding landscapes. Monczor *et al.* showed that 3 GR ligands, dexamethasone, RU486, and cyproterone, exhibited unique dose-dependent gene transcriptional responses for three classical GR-target genes (*GILZ*, *SLC19A2*, and *THBD*) (130). Chromatin immunoprecipitation analysis showed that the location and proximity of the GREs in the promoters of GR target genes only explained a portion of the ligand-specific gene transcriptional response (131). This point is illustrated by Wang *et al.*, where GR shows no preference for GRE sequences in the dual luciferase assay in the absence of coregulator proteins (131). Using chromatin immunoprecipitation, they showed that GR has subsets of primary and secondary-regulated pathways that drive the binding of GR to certain GREs (131). They hypothesize that the differences in transcriptional responses are likely due to the presence of coregulator proteins. A continuation of this concept is demonstrated by Monczor *et al.*, who quantified the distinct coregulator profiles of purified GR protein stimulated by dexamethasone, RU486, and cyproterone (130). The GR-dexamethasone coregulator profile had significantly higher signal intensity values, indicating stronger coregulator binding than RU486 and cyproterone counterparts (130). They identified that the coregulator NR0B1 (nuclear receptor subfamily 0 group B member 1) had the most difference in binding affinity for GR, with dexamethasone causing more binding and RU486 having less (130). Using siRNA, they found that inhibiting NR0B1 from binding GR, that its target gene *SLPI* (secretory leukocyte peptidase inhibitor) was only one affected when A549 cells were treated with dexamethasone, but this was not observed with RU486 (130). The Fitzsimons group also identified several coregulators regulated by ligand-specific GR interactions (130). The coregulator motif CBP_57_80 (CREB-binding protein) binds to the GR coregulator complex when activated by dexamethasone (130). When stimulated by cyproterone, the same motif dissociates from GR, and there is no interaction when RU486 antagonizes GR (130). In tandem with RNA-seq data that show unique transcriptional patterns for each GR ligand, the Fitzsimons group has utilized the NHR PamChip technology to demonstrate the contribution of coregulators in mediating ligand-specific pathways (130).

Other groups have utilized the NHR PamChip technology to understand the effects of ER-activating compounds that act as endocrine disruptors. An endocrine disruptor interferes with normal hormone signaling and function of the endocrine system. Endocrine disruptors may affect steroid receptor signaling, causing infertility, metabolic syndrome, cancer, and other conditions (132). Aarts *et al.* screened 25 compounds using purified ERα protein and the NHR PamChip assay to determine the ligand-specific coregulator profiles (133). They found inconsistencies in the ERα coregulator profiles of endocrine disruptors when compared to known ERα agonists, such as a robust binding to NCOA1 with ERα when activated by 17β-estradiol, but not the endocrine disruptor dibutyl phthalate (133). These data suggest an alternative role of endocrine disruptors in regulating the coregulator binding of nuclear receptors. Houtman *et al.* showed that the phosphorylation of ERα at serine 305 (Ser305) dose dependently modifies the ability of NCOA1 to interact with ERα when stimulated with 17-β-estradiol in U2OS cells (134). They demonstrated Ser305 phosphorylation decreased interactions with NCOA1 with 17-β-estradiol treatment but found increased dose-dependent binding when U2OS cells were treated with tamoxifen (134). These findings are consistent with others, where another group found that Ser305 phosphorylation of ERα induces a unique interaction with NCOA1 (135). This study suggests that the Ser305 phosphorylation of ERα is positively correlated with tamoxifen resistance and could be partially due to the interaction of coregulator proteins (134).

Perera *et al.* utilized the NHR PamChip technology to study endocrine disruptors and their effects on AR (136). Using purified AR protein, they quantified the AR coregulator profile for the endocrine disruptors bisphenol A (BPA), bisphenol AF (BPAF), and bisphenol S against known AR agonist and antagonist, R1881 and cyproterone acetate (CPA) (136). Hierarchical clustering of the coregulator signal intensities revealed that BPA and BPAF shared a more similar profile to CPA for several coregulator motifs corresponding to CNOT and NCOR1, indicating that BPA and BPAF modulate the coregulator interactions of AR as antagonists (136). Their analysis showed that the androgen receptor (ANDR_10_32) motif was the only shared positively interacting coregulator when comparing R1881 and BPA without any overlap for negatively interacting coregulators (136). BPA shared several positively interacting coregulators compared to CPA, including IKBB, CNOT1, and DHX30 motifs, which are thought to suppress transcription (137, 138). BPA and CPA also shared negatively interacting coregulators, including NCOR1, NR0B1, and NRIP1 (136), which have coactivation and repressive functions (139, 140, 141). Perera *et al.* indicated that this could be partly explained by the detrimental effects of BPA associated with endocrine disruption (136). They also determined that BPA analogs could modify the AF-2 coregulator binding site within the AR protein, specifically the H3, H5, and H12 alpha-helices of the AR structure that allow for coregulator binding (136). Using molecular docking simulations, they found that BPA has a relatively high affinity for the LBD, AF-2, and binding function 3 sites on AR, which all modulated the binding of other coregulatory proteins (136). The relatively high binding affinity of BPA suggests that many combinations of coregulator complexes can arise from BPA exposure, explaining some of the difficulty of studying the large class of endocrine disruptors. This also demonstrates using the NHR PamChip to better understand how chemicals might lead to disease and improve therapeutic development.

iii. NHR PamChip studies on other nuclear receptors

Koppen *et al.* used the NHR PamChip technology to quantify the PPARγ coregulator interactome with rosiglitazone and GW9662 (142), two known PPARγ ligands (143, 144). PPARγ is an attractive drug target for obesity and diabetes for its role in adipogenesis and fat storage (145). However, PPARγ agonists are associated with several adverse side effects (143), which might be from diverse coregulator interactions. Using the NHR PamChip technology, Koppen and colleagues found that thyroid hormone receptor-interacting protein 3 (TRIP3) and NCOR1 interacted only with rosiglitazone-activated PPARγ but not PPARγ antagonist GW9662 (142). The authors determined that NCOR1 and TRIP3 have an inverse binding to PPARγ when it is activated by rosiglitazone but not with GW9662, suggesting differential regulation (142). They also identify TRIP3 as a novel regulator of adipocyte differentiation using the NHR PamChip technology, providing evidence for its use in identifying disease mechanisms (142).

Gordon *et al.* characterized the PPARα coregulator interactome for three PPARα ligands using purified protein to identify ligand-specific regulation of fat-burning pathways (146). When activated by bilirubin, fenofibrate, or WY 14,643, purified human PPARα LBD displayed the strongest interaction with coregulators with WY-14,643 treatment (146). When the mouse full-length PPARα purified protein was activated by bilirubin, it displayed the strongest interaction with coregulators, with WY-14,643 having the weakest interaction signature profile (146). They also measured the coregulator profile of PPARα in the white adipose tissue of diet-induced obese mice treated with bilirubin nanoparticles. The results showed an increased interaction of PPARα with NCOA1, NCOA2, and NCOA3 and decreased interactions with several NRIP1 motifs, as well as NCOR1 and NCOR2 (146). NRIP1 has been shown to repress PPARα and increase PPARγ to control fat accumulation and mediate mitochondrial function (147). The bilirubin nanoparticles also increased mitochondrial function in white adipose tissue, attributed to the restructuring of corepressors to coactivators to PPARα with bilirubin-bound (146). This technology was essential in identifying bilirubin as a hormone (148, 149, 150, 151, 152). It further enhances the therapeutic potential of drug development and targeting coregulator profiles to modulate specific disease-causing pathways.

## Conclusions

A deeper understanding of the relationships of coregulator interactomes with steroid receptor complexes provides insight into pathways induced by ligand-specific pathway activation and, more importantly, targetable proteins to drive specific pathways. Steroid receptor signaling mechanisms are tightly controlled by a protein complex that regulates physiological functions. Advanced technology like the PamGene PamStation offers avenues to investigate the binding landscape of proteins in a disease or better understand the mechanisms of action for a drug–steroid receptor interaction. This opens more possibilities for drug development in targeting these signaling mechanisms. There is still much to be learned about the coregulator proteins and how the regulate gene function through nuclear receptors.

## Conflicts of interest

The authors declare that they have no conflicts of interest with the contents of this article.
